# A Novel Mutation of *AMHR2* in Two Siblings with Persistent Müllerian Duct Syndrome

**DOI:** 10.4274/jcrpe.0013

**Published:** 2018-11-29

**Authors:** Edip Unal, Ruken Yıldırım, Suat Tekin, Vasfiye Demir, Hüseyin Onay, Yusuf Kenan Haspolat

**Affiliations:** 1 Dicle University Faculty of Medicine, Department of Pediatric Endocrinology, Diyarbakır, Turkey; 2 Diyarbakır Children’s Hospital, Clinic of Pediatric Endocrinology, Diyarbakır, Turkey; 3 Dicle University Faculty of Medicine, Department of Pediatrics, Diyarbakır, Turkey; 4 Kocaköy Family Health Center, Diyarbakır, Turkey; 5 Ege University Faculty of Medicine, Department of Medical Genetics, İzmir, Turkey

**Keywords:** Undescended testis, anti-Müllerian hormone, persistent Müllerian Duct syndrome

## Abstract

Persistent Müllerian Duct syndrome (PMDS) develops due to deficiency of anti-Müllerian hormone (AMH) or insensitivity of target organs to AMH in individuals with 46,XY karyotype. PMDS is characterized by normal male phenotype of external genitals, associated with persistence of Müllerian structures. This report includes the presentation of a 2.5 year old male patient due to bilateral undescended testis. His karyotype was 46,XY. The increase in testosterone following human chorionic gonadotropin stimulation test was normal. The patient was referred to our clinic after uterine, fallopian tube and vaginal remnants were recognized during the orchiopexy surgery. The family reported that the eight year old elder brother of the patient was operated on for right inguinal hernia and left undescended testis at the age of one year. A right transverse testicular ectopia was found in the elder brother. Both cases had normal AMH levels. *AMHR2* gene was analyzed and a homozygous NM_020547.3:c.233-1G>A mutation was found that was not identified previously. In conclusion, we determined a novel mutation in the *AMHR2* gene that was identified for the first time. This presented with different phenotypes in two siblings.

**What is already known on this topic?**

Persistent Müllerian Duct syndrome develops due to deficiency of anti-Müllerian hormone (AMH) or AMH receptor resistance in individuals with 46,XY karyotype. The condition is characterized by a penis of normal length in association with unilateral or bilateral undescended testes and persistence of müllerian structures in individuals with 46,XY karyotype.

**What this study adds?**

A novel homozygous mutation in the AMHR2 gene was found in two siblings. These siblings were phenotypically different, suggesting that this mutation may present with a variable clinical picture.

## Introduction

Persistent Müllerian Duct syndrome (PMDS) is a rare disorder of 46,XY sex development. The condition is characterized by a penis of normal length in association with unilateral or bilateral undescended testis and persistence of Müllerian structures in individuals with 46,XY karyotype. PMDS develops mostly due to deficiency of anti-Müllerian hormone (AMH) or insensitivity of target organs to AMH. Mutations of either the *AMH* or *AMHR2* gene have been detected in 88% of cases (1). PMDS shows an autosomal recessive inheritance and its incidence is not clearly known. However, published numbers of cases have increased, due to cryptorchidism being investigated at earlier stages of life, laparoscopic examination being included in routine clinical work-up and surgeons being more aware of this condition in comparison to the past (1).

In this study, we present two siblings with homozygous NM_020547.3:c.233-1G>A mutation that is identified for the first time in the *AMHR2* gene. One sibling presented with bilateral undescended testis, while the other presented with transverse testicular ectopia.

## Case Reports

### Case 1

This 2.5 years old male patient presented to our outpatient clinic with bilateral undescended testis. Family history is notable for 3^rd^ degree consanguineous marriage in the parents. It is understood that bilateral, undescended testes were recognized by the family immediately after the birth, but the family did not visit a doctor. At presentation the patient had a weight of 13.5 kg [standard deviation score (SDS): -0.18] and a height of 94.0 cm (SDS score: 0.36). Stretched penile length was 4 cm. The testes could not be palpated bilaterally. A pelvic ultrasound detected a formation, suggestive of testis in the proximal segment of the inguinal canal, bilaterally. These structures were 7x5x7 mm in size on the right side and 7x5x9 mm in size on the left side. A uterus, Fallopian tubes or ovaries could not be visualized. Laboratory tests revealed a follicle stimulating hormone (FSH) concentration of 1.2 mIU/mL, luteinizing hormone (LH) concentration of 0.1 mIU/mL and total testosterone of 0.03 ng/mL. Concentration of 17-hydroxyprogesterone was 0.48 ng/mL and AMH: 35.1 ng/mL (normal range: 5-265 ng/mL). As the patient had bilateral undescended testes and the hormone profile was prepubertal, a human chorionic gonadotropin stimulation test was performed and testosterone response was normal. The patient was referred to the pediatric surgery clinic for orchiopexy. Rudimentary uterine, fallopian tube and vaginal remnants were seen at the orchiopexy operation. It was also reported that bilateral gonads resembling testes were found and biopsies were taken. The patient was referred to our outpatient clinic again due to the presence of these Müllerian structures. The biopsy specimens were consistent with bilateral testicular tissue. In the light of these findings, a diagnosis of PMDS was considered. Since AMH level was normal, *AMHR2* gene mutation was considered.

**Figure 1:**
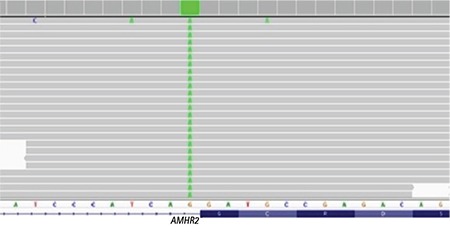
Homozygous mutation (NM_020547.3:c.233-1G>A) in intron 2 of the *AMHR2* gene

*AMHR2* gene mutation analysis was performed by sequencing of the coding exons and the exon-intron boundaries of the genes. Genomic DNA was isolated from peripheral blood cells with QIAGEN (Maryland, USA) DNA Blood Mini Kit according to the protocol provided with the kit. Sequencing was performed with Miseq V2 chemistry on MiSeq instrument (Illumina California, USA). Analysis was performed with Integrative Genomics Viewer software. Genetic analysis revealed a homozygous NM_020547.3:c.233-1G>A mutation in the *AMHR2* gene (Figure 1). This mutation has not been identified in databases previously. Analyses made with MutationTaster, and splicing modeling software (NNSplice, GeneSplicer and SpliceSiteFinder) showed that the mutation can cause the condition. According to the American College of Medical Genetics and Genomics 2015 criteria, this mutation was classified as “Pathogenic” (2).

### Case 2

After case 1 was diagnosed with PMDS, the elder sibling (eight years old) presented due to left undescended testis. From the patient history it was learned that the family had noted a swelling in the right groin when the child was two months old and that the child was operated on by a pediatric surgeon at age one year. Right inguinal hernia and presence of both testes in the right scrotum were reported after the surgery. Right inguinal hernia was repaired, but Müllerian structures were not mentioned in the operative note. At presentation the patient weighed 23.5 kg (SDS: -0.65) had a height of 124 cm (SDS: -0.54). The left testis could not be palpated, but both testes were palpated in the right scrotum. The stretched penile length was 5 cm. The scrotal ultrasound was consistent with the presence of two testes in the right scrotal cavity, measuring 15x7 mm and 17x7 mm respectively. A structure consistent with a uterus, measuring 8x4 mm in size, was found posterior to the urinary bladder. FSH level was 0.72 mIU/mL, LH: 0.1 mIU/mL, total testosterone: 0.03 ng/mL, AMH: 35.1 ng/mL (normal range: 3.2-182.4). Since AMH level was normal, *AMHR2* gene mutation was considered and a homozygous NM_020547.3:c.233-1G>A mutation was found in the *AMHR2* gene in the genetic analysis.

Written informed consent was obtained from the patient’s family for publication of this report.

## Discussion

AMH is synthesized by immature Sertoli cells in men and ovarian granulosa cells in women. It is responsible for total regression of Müllerian structures in week 10 of fetal development in the male fetus. External genitalia are completely normal in men with AMH deficiency. However, AMH deficiency causes persistence of Müllerian structures along with testes and male excretory ducts (1,3). PMDS usually originates from gene mutations in *AMH* or *AMHR2* (1,4). It is recognized during conventional surgery or laparoscopic examination of undescended testis alone or in combination with inguinal hernia (1). PMDS has three main clinical presentations:

1. Bilateral cryptorchidism. This presentation accounts for approximately 55% of AMH pathway mutations and 86% of idiopathic cases;

2. Unilateral cryptorchidism. A testis and the accompanying fallopian tube and uterus cause an inguinal hernia. This presentation is known as “hernia uteri inguinalis”. This presentation accounts for approximately 20% of AMH pathway mutations and 14% of idiopathic cases;

3. Transverse testicular ectopia. This term refers to unilateral herniation of both testes and a part of the Müllerian structures through the processus vaginalis. This condition is the most specific anatomic situation of PMDS and it is found in 25% of cases with *AMH* or *AMHR2* gene mutation. However, it is never seen in idiopathic cases (5). Our first case was diagnosed with this condition, after Müllerian structures were recognized during surgical treatment of bilateral undescended testis.

AMH is a member of the transforming growth factor-β family. It contains fixed exons and it is 2.8 kb long (6). Cohen-Haguenauer et al (7) determined that the *AMH* gene is located in the short arm of chromosome 19 (p13.3). It was reported that the *AMHR2* gene is located on the long arm of chromosome 12 and that it contains 11 exons (8). Picard et al (1) conducted a study of 157 families with PMDS from 1990 to 2016 and they found mutations of *AMH* or *AMHR2* genes in 88% of the cases. The same study demonstrated 64 different mutations in the *AMH* gene in 80 families and the authors found that mutations are more commonly located in Exon 1, 2 and 5. Similarly, *AMHR2* gene mutations were discovered in 75 families in 58 different alleles. No mutation was found in the AMH or *AMHR2* genes in 12% of the cases and these are referred to as idiopathic PMDS (1). Serum *AMH* levels are undetectable or low in *AMH* gene mutations, while normal or high when *AMHR2* mutations are present (4,9). Since serum *AMH* levels were normal in our patient, *AMHR2* gene mutation was considered and this diagnosis was made. There is no significant anatomic difference between patients with *AMH* or *AMHR2* gene mutations. Previous studies demonstrated that the position of testes and that of Müllerian structures may vary between siblings with PMDS and with the same mutation (10). Our study showed that a mutation causes bilateral undescended testis in one patient and transverse testicular ectopia in the sibling.

Recently, early orchiopexy has been recommended, if and whenever possible, in order to prevent damage to the germ cells in patients with cryptorchidism. Previously, it was estimated that the incidence of testicular cancer in PMDS was not higher than that in cases with cryptorchidism and that the incidence was around 18% (11). However, Picard et al (1) showed that unilateral or bilateral malignant testicular degeneration develops in 33% of patients with PMDS at ages 18 or above and stated that the most common malignant degeneration is seminoma. Malignant degeneration of Müllerian derivatives is less common. Farikullah et al (12) have detected degeneration of Müllerian structures releated to PMDS with only in 3 cases in their study.

The most common complication of PMDS is infertility. Although fertility is rare in PMDS, it is possible if at least one testis is present and if the excretory ducts are intact (1). A comprehensive literature review showed that 19% of adult patients have one or more children (1). Farag (13) reported the rate of fertile patients as 11%. On the other hand, there are many reported cases of infertility and azoospermia. Late orchidopexy, damage of testis and vas deferens during surgery and abnormal anatomic connection of testes to the excretory ducts are some of the causes of infertility (13,14,15). The testes are usually not properly connected to the male excretory ducts due to aplasia at the upper part of the vas deferens and epididymis or absence of a connection between the testis and the epididymis (16).

In conclusion, PMDS is a rare condition that is usually seen in men who present with cryptorchidism and/or inguinal hernia. It should be diagnosed early for both protection of fertility and for prevention of potential malignant degeneration. Considering the possibility of damage to the vas deferense and testis during surgical procedures, the patient should always be referred to experienced surgeons.
